# Clinical presentations, management outcomes, and diagnostic dilemma in *Kocuria* endophthalmitis

**DOI:** 10.1186/s12348-018-0163-6

**Published:** 2018-11-20

**Authors:** Vivek Pravin Dave, Joveeta Joseph, Avinash Pathengay, Rajeev R. Pappuru

**Affiliations:** 1 Smt. Kanuri Santhamma Center for Vitreoretinal Diseases, Kallam Anji Reddy Campus, LV Prasad Eye Institute, Hyderabad, Telangana 500034 India; 2 Jhaveri Microbiology Center, Brien Holden Eye Research Center, LV Prasad Eye Institute, Hyderabad, India; 3 Retina and Uveitis Department, GMR Varalaxmi Campus, LV Prasad Eye Institute, Hanumanthawaka Chowk, Visakhapatnam, Andhra Pradesh 530040 India

**Keywords:** Endophthalmitis, *Kocuria*, Coagulase-negative *Staphylococci*

## Abstract

**Aim:**

To describe the clinical presentations and management outcomes of *Kocuria* endophthalmitis and discuss diagnostic dilemmas

**Design:**

Retrospective interventional comparative case series

**Intervention:**

Eight unilateral cases with culture-proven *Kocuria* endophthalmitis from January 2013 to December 2017 underwent vitrectomy/vitreous biopsy and intravitreal antibiotic with or without additional procedures. The undiluted vitreous was subjected to microbiologic evaluation.

**Main outcome measures:**

The mean age at presentation, etiology, number of interventions, interval between inciting event and presentation, type of intravitreal antibiotic used, and anatomic and functional outcomes were reported. A favorable anatomic outcome was defined as preservation of the globe, absence of hypotony, attached retina, and absence of active inflammation at the last visit.

**Results:**

In the current series, there were five males and three females. The mean age at presentation was 31 ± 17.44 years (median 30 years). The inciting event was open globe injury in five cases and one case each following cataract surgery, microbial keratitis, and endogenous cause. Visual acuity was ≥ 20/400 in one case at presentation and ≥ 20/400 in three cases at the last visit. The species identified by Vitek 2, included *Kocuria kristinae* in three, *K. rosea* in four and *K. varians* in one. Follow-up period was 8.25 ± 8.24 months (median 6.5). Six cases (75%) had complete resolution of infection and inflammation at the last visit. Anatomic success was achieved in 75%.

**Conclusions:**

*Kocuria* is a relatively rare cause of endophthalmitis often misdiagnosed as *Staphylococcal* endophthalmitis. Clinical presentation can be variable but favorable antibiotic susceptibility and appropriate timely management can result in acceptable visual and anatomic outcomes.

## Summary statement

*Kocuria* species can mimic other coagulase-negative *Staphylococci* and cause diagnostic dilemma. Rarely, *Kocuria* can be a cause of endophthalmitis. In the current communication, we describe our experience of diagnosing and managing endophthalmitis due to *Kocuria* spp.

## Introduction

*Kocuria* are gram-positive coccoid bacteria of the family *Micrococcaceae* (order *Actinomycetales*, class *Actinobacteria*). They are found as tetrads or irregular clusters and are biochemically catalase-positive and coagulase-negative. *Kocuria* infections over the years have been reported largely as systemic infections in immunocompromised hosts [1, 2]. Though *Kocuria* has been known to be a part of the normal microbiota, it can cause infections like peritonitis, endocarditis, cholecystitis, pneumonitis, and urinary catheter-related infections [3, 4–16]. *Kocuria* infection prevalence could be falsely low as phenotypic assays used for organism identification often misdiagnose *Kocuria* as *Staphylococci* [17]. In the current communication, we report the presentations, management outcomes, and diagnostic dilemma in a series of *Kocuria* endophthalmitis treated at our center.

## Methods

This was a retrospective consecutive interventional case series. This was a retrospective chart review of cases with culture-positive *Kocuria* endophthalmitis. We included all cases with culture-positive *Kocuria* endophthalmitis from January 2013 to March 2018 at our tertiary eye care center. The study protocol was approved by the institutional review board. The medical records of the patients were retrieved from the medical records department and the microbiology laboratory database. The collected demographic information included the etiology of the infection, interval from the inciting event to the start of symptoms, interval between the start of symptoms and treatment intervention, comorbid systemic illnesses, visual acuity at presentation, detailed biomicroscopic examination including information on media clarity on indirect ophthalmoscopy, retinal examination, ultrasound findings, treatment provided, and final functional and anatomic outcomes.

### Management protocol

Management of the patients depended on their clinical presentation. Cases with milder presentations or with poor corneal clarity underwent vitreous biopsy. Those with severe vitritis as confirmed clinically or on B scan underwent pars plana vitrectomy wherever the corneal clarity allowed the same. All patients were initially empirically treated with intravitreal vancomycin (1 mg/0.1 ml) and ceftazidime (2.25 mg/0.1 ml) after collecting the vitreous sample. The vitreous sample was collected from the mid-vitreous cavity with a 2-cc syringe plugged into the suction tubing of the vitreous cutter on cutting mode and before opening the infusion fluid. Further injections were based on the culture report. If no response was noted, a repeat intravitreal injection or a pars plana vitrectomy was done after 48–72 h depending on the corneal clarity. In some patients, additionally, anterior chamber fluid and the explanted intraocular lens were also processed. Vitreous samples were transported to the microbiology laboratory immediately and examined by direct microscopy (Calcofluor white, Gram, Giemsa stains) and cultured on appropriate bacterial (aerobic and anaerobic) and fungal media. All media were incubated at 37 °C for 1 week except SDA which was incubated at 25 °C. Only significant culture results were considered. Growth on two or more media or confluent growth on at least one solid medium at the site of inoculation or growth on one medium with consistent direct microscopy result was defined as a significant positive culture. The culture isolates were further identified by biochemical tests and Vitek 2 (Biomerieux, France). The organisms were identified using the Vitek 2 ID GPC gram-positive identification card which uses a fluorogenic methodology for organism identification. After the initial surgical intervention, the patients received topical antibiotics like ciprofloxacin 0.3%, cycloplegics, and topical steroids. Systemically, the patients were administered oral ciprofloxacin 750 mg twice a day empirically.

### Outcome definition

The outcome at the last visit was evaluated in terms of anatomic and functional outcome. A favorable anatomic outcome was defined as preservation of the globe, absence of hypotony, attached retina, and absence of active inflammation at the last visit. A favorable functional outcome was defined as an attached retina with a vision of ≥ 20/400 at the last visit.

## Results

The current case series included eight eyes of eight patients. There were five males and three females. The mean age at presentation was 31 ± 17.44 years (median 30 years). None of the patients had any systemic illness and all were immunocompetent. The inciting event was open globe injury in five cases and cataract surgery, microbial keratitis, and endogenous cause in one case each. The case with microbial keratitis had a corneal ulcer perforation and at presentation already had a primary procedure of a tissue adhesive application done elsewhere.

The ulcer finally resolved, and at the last visit, the patient showed a vascularized corneal scar. Of the three patients with post-trauma endophthalmitis who presented with a corneal tear, all show a favorable anatomic outcome while two also had a favorable visual outcome. One case presenting with endogenous endophthalmitis developed a quiet anterior chamber but had persistent vitritis at the last visit. The eye with an open globe injury with scleral involvement developed a recurrent retinal detachment with an overall poor anatomic and functional outcome. The clinical features, the type of intervention, the number of injections, and the medications used are summarized in Table 1. The presenting visual acuity was favorable in one case, while at the final visit, it was favorable in three cases. At the last visit, endophthalmitis was noted to be resolved in six cases. The tissue sample that tested positive for *Kocuria* sp. varied with the cases and is summarized in Table 2.

**Table 1:** Clinical features and management outcomes of *Kocuria* endophthalmitis

Case no.	Gender	Age	Presenting vision	Setting of infection	Anterior chamber findings	Posterior segment findings	Interval between symptom start and presentation (days)	Initial intervention	Number of repeat intravitreal antibiotic injections	Follow-up in months	Final visual acuity	Final anatomic outcome	Final visual outcome	Cause of low final vision	Species isolated
1	M	57	HM	Post keratitis	Corneal edema, perforation with TABCL in situ	No view	30	Vitreous biopsy, IOAB	2	5	HM	F	UF	Corneal scar	*K. varians*
2	F	34	20/400	Post trauma	Corneal tear, traumatic cataract	No view	1	CTR, vitreous biopsy, IOAB	5	11	20/2400	F	UF	Corneal scar	*K. rosea*
3	M	26	PL	Post trauma	Scleral tear, hypopyon, cataract	No view	2	STR, PPV, IOAB, PPL	3	8	HM	UF	UF	BSK, recurrent RD	*K. rosea*
4	M	36	PL	Post trauma	Cornedema, hypopyon	No view	20	PPV, PPL, IOAB	3	3	HM	UF	UF	Recurrent RD	*K. kristinae*
5	M	7	PL	Post trauma	Corneal tear, hypopyon	No view	1	CTR, vit biopsy, IOAB	2	27	20/60	F	F	Central corneal scar	*K. kristinae*
6	M	18	CFCF	Post trauma	Corneal tear, hypopyon	No view	1	CTR, vit biopsy, IOAB	2	8	20/60	F	F	Central corneal scar	*K. kristinae*
7	F	18	PL	Endogenous	Corneal edema, hypopyon	No view	2	PPL, PPV, IOAB	2	2	HM	F	UF	Recurrent vitritis	*K. rosea*
8	F	52	CFCF	Postoperative	Corneal edema, AC cells	Retina attached, disc seen hazily	4	PPV, IOAB	1	2	20/80	F	F	CME	*K. rosea*

*TABCL* tissue adhesive with bandage contact lens, *IOAB* intraocular antibiotics, *CTR* corneal tear repair, *STR* scleral tear repair, *PPV* pars plana vitrectomy, *PPL* pars plana lensectomy, *F* favorable, *UF* unfavorable

**Table 2:** Smear and culture positivity for various tissue samples in the current series

Case	AC smear	AC culture	Vitreous smear	Vitreous culture
1	−	+	−	−
2	−	−	+	+
3	−	−	−	+
4	−	−	−	+
5	−	−	−	+
6	−	+*	+	+
7	+	−	+	+^#^
8	−	−	+	+

*AC* anterior chamber

*AC culture-positive for *Aspergillus niger* not for *Kocuria*

^#^Culture-positive for *Kocuria* and also for *Streptococcus* species

**Fig. 1:**
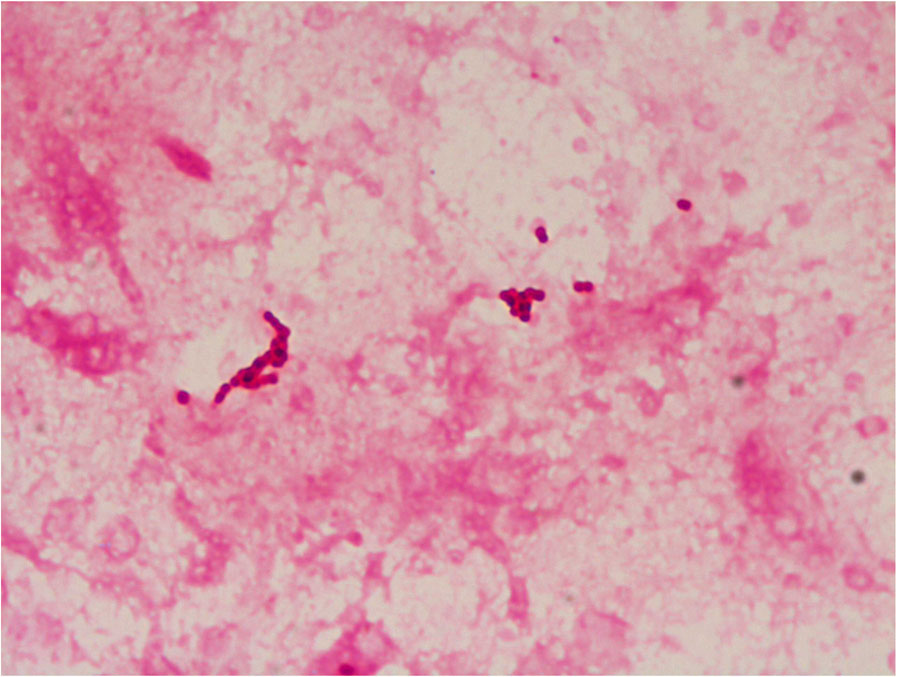
Gram stain (× 100) of vitreous sample (case 2) showing gram-positive cocci in pair and short-chain 0-6/oil immersion field

**Fig. 2:**
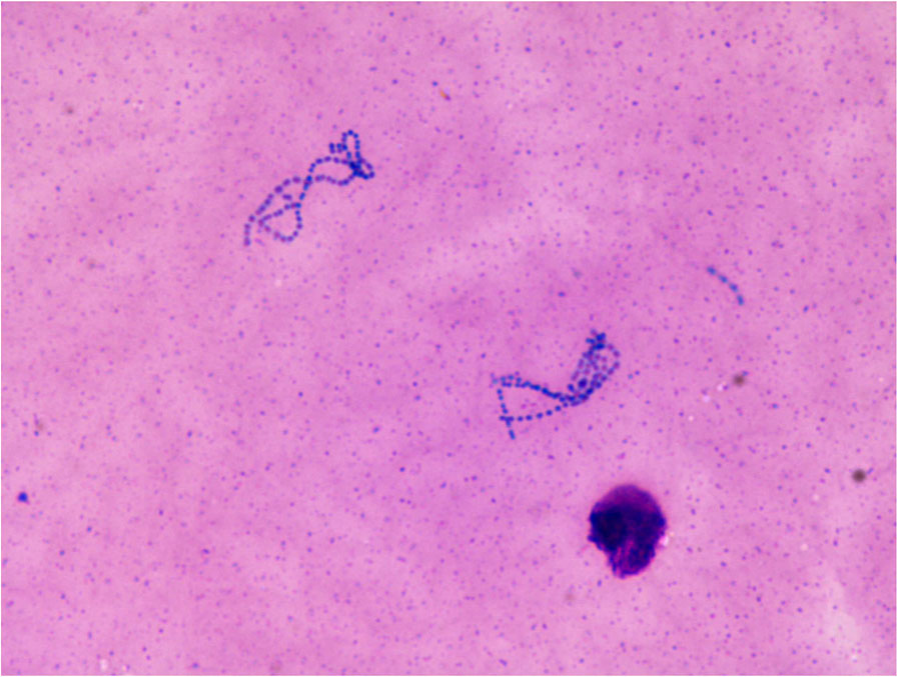
Giemsa stain (× 100) of vitreous sample (case 7) showing gram-positive cocci in chains 0-plenty/oil immersion field

**Fig. 3:**
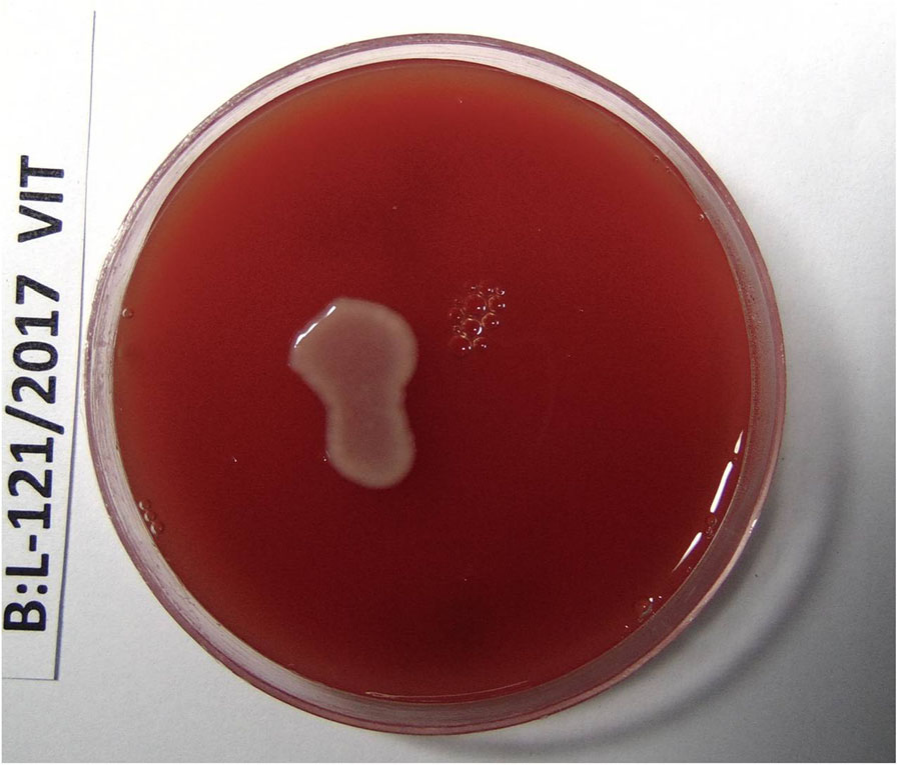
Culture of vitreous sample (case 2) showing large smooth convex cream-colored colonies on blood agar after 48-h incubation which was identified on Vitek 2 as *Kocuria rosea*

**Fig. 4:**
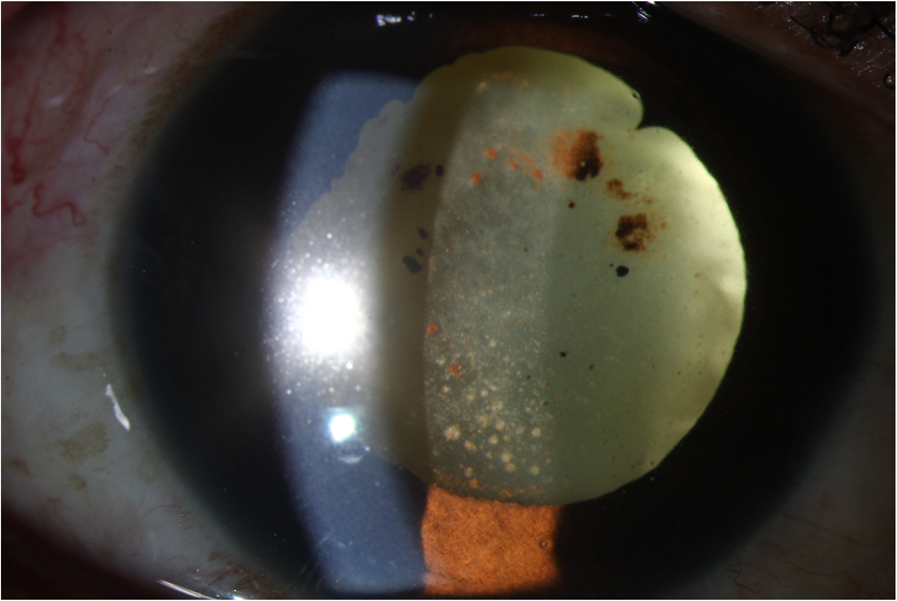
Preoperative slit lamp photograph of endogenous Kocuria endophthalmitis case

**Fig. 5:**
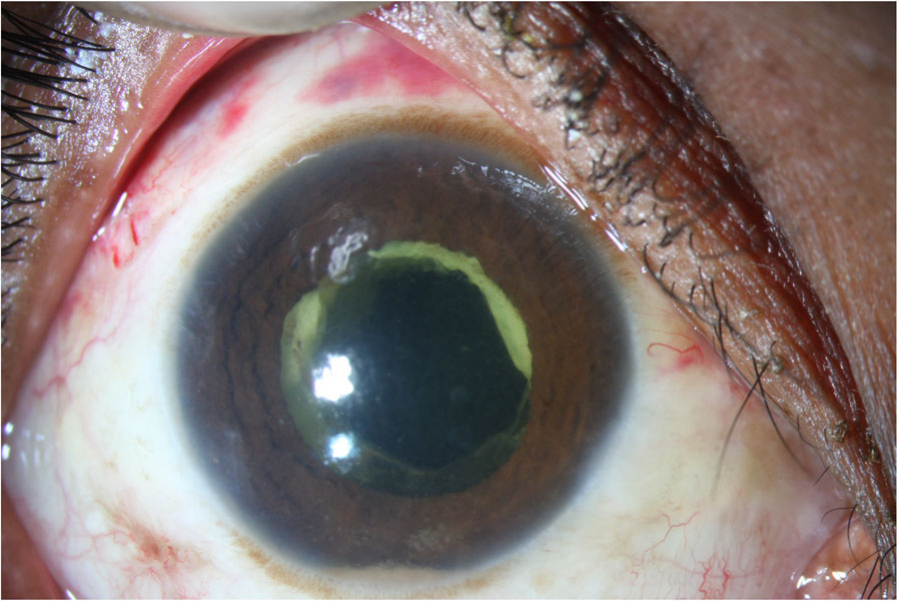
Postoperative slit lamp photograph of the endogenous case following pars plana vitrectomy and lensectomy

**Fig. 6:**
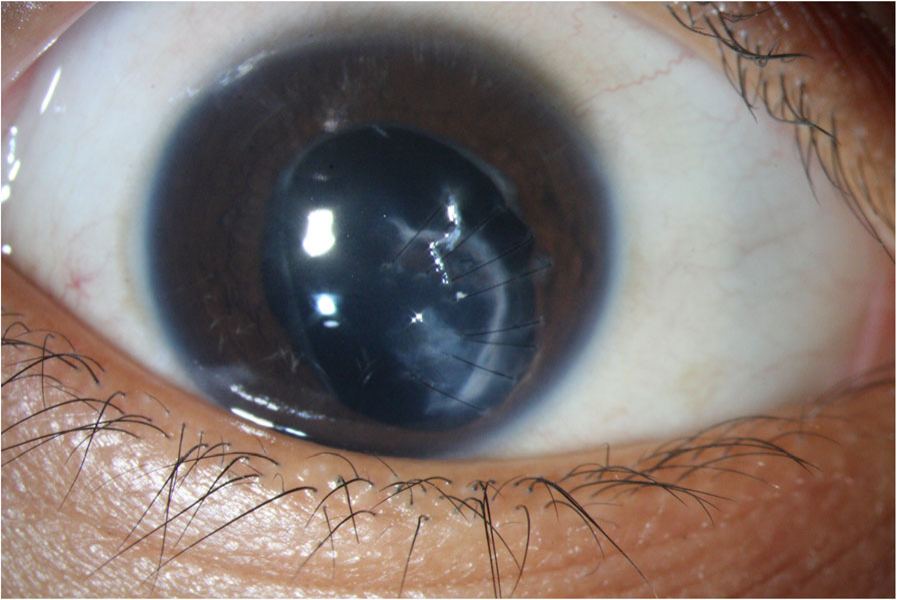
Postoperative photograph of resolved *Kocuria* endophthalmitis following open globe trauma with a repaired corneal laceration

**Fig. 7:**
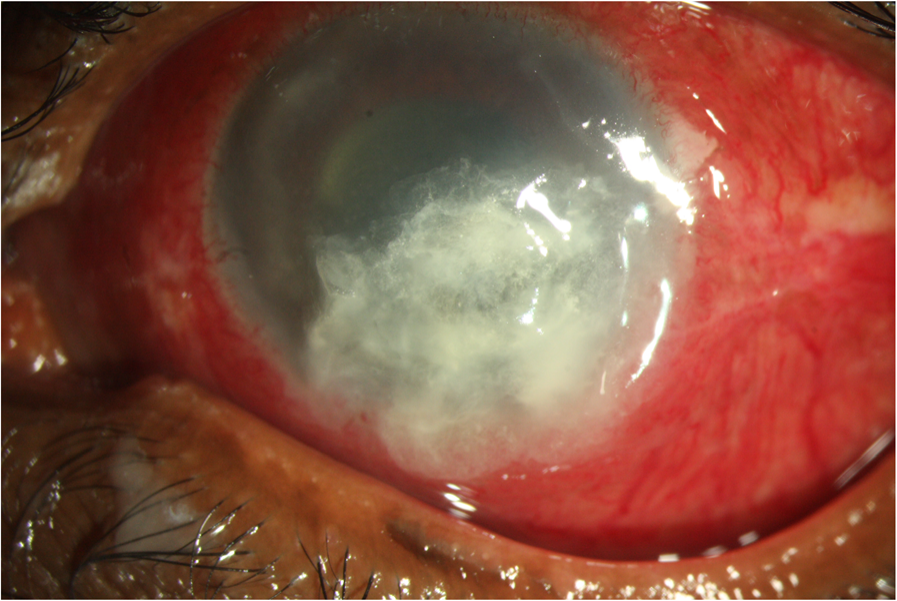
Preoperative slit lamp photograph of a case of *Kocuria* endophthalmitis post perforated corneal ulcer with tissue adhesive in situ

The direct microscopy of the vitreous fluid showed the presence of gram-positive cocci in pairs, groups, and chains (Figs. 1 and 2) under gram stain in four of eight cases in our series. Presence of *Kocuria* sp. was confirmed by growth of large, cream-colored colonies on chocolate and/or blood agar (Fig. 3) from the vitreous fluid in seven of eight cases and from AC Tap in one of eight cases. All of these isolates were catalase positive and coagulase negative. Vitek 2 identification revealed the presence of *Kocuria rosea* in four cases, *K. kristinae* in three cases, and *K. varians* in one case using the GP card*.* Overall antibiotic susceptibility to most commonly used antibiotics was found to be good (Table 3). Figures 4, 5, 6, and 7 depict clinical features at presentation and final visits of a few cases.

**Table 3:** Case-wise isolated *Kocuria* species and antibiotic susceptibility

Case number	Species	Amikacin	Cefazolin	Chloramphenicol	Ciprofloxacin	Moxifloxacin	Vancomycin	Cefuroxime
1	*K. varians*	+	+	+	+	+	+	+
2	*K. rosea*	+	+	+	+	+	+	+
3	*K. rosea*	+	+	+	+	+	+	+
4	*K. kristinae*	−	−	+	+	+	+	+
5	*K. kristinae*	+	+	+	+	+	+	+
6	*K. kristinae*	+	+	+	+	+	+	+
7	*K. rosea*	−	+	+	+	+	+	+
8	*K. rosea*	+	+	+	+	+	+	+

## Discussion

The current report describes the outcome of a series of cases of *Kocuria* endophthalmitis. In a retrospective study from China describing the causative organisms in infectious endophthalmitis, 1/330 cases reported was reported as *Kocuria* [17]. While cases of *Kocuria* keratitis have been reported previously with variable visual outcome [18, 19], this is the first detailed description of endophthalmitis caused by *Kocuria* sp. *Kocuria* is a member of the Micrococcaceae family and currently includes 18 species [1, 2, 20]. Though it is ubiquitous in nature and freely found as normal skin and oral flora in humans, only 5/18 known species are known to be opportunistic pathogens [1]. The diagnostic difficulties in *Kocuria* identification lie in the fact that the phenotypic assays used for microbe identification often misdiagnose *Kocuria* as coagulase-negative *Staphylococci* [21]. Further diagnostic difficulty occurs due to a differential behavior of *Kocuria* in vitro and in vivo especially in stress conditions [22, 23]. Pathological strains are known to be more phenotypically variable then saprophytic strains [24–27]. Identification of *Kocuria* spp. remains elusive because most clinical microbiology laboratories have limited or no access to advanced molecular techniques. Laboratory identification of *Kocuria* spp. can be made conventionally only on a high index of suspicion. Properties such as morphological variability between these bacteria and other similar gram-positive cocci, as well as biochemical properties including the antimicrobial susceptibility patterns against selective antibiotics, could be used to presumptively identify *Kocuria* spp. Susceptibility towards bacitracin and lysozyme and resistance to nitrofurantoin, furazolidone, and lysostaphin can be used to separate this bacterium from *Staphylococci*. This however is not commonly done in routine work-ups. Increase in time-dependent pigmentation of the colonies can also lead to a diagnosis of *Kocuria* as the typical pigmentation of these colonies increase with time especially after a culture period of 48 h [1]. The introduction of the Vitek 2 GP-card database allows for a better identification of *Kocuria* in the recent times [28]. Though there are reports of coagulase-negative *Staphylococci* being wrongly identified as *Kocuria* by the Vitek 2 system [21], other more recent studies indicate a good reliability of the Vitek 2 system [29, 30]. Our case series includes cases that have been identified by Vitek 2 system alone. The clinical features of the cases in this series included findings like open globe trauma, hypopyon, vitritis, repeat intravitreal antibiotic injections, and an overall fair resolution of the vitritis. These clinical features largely mimic those of endophthalmitis following a *Staphylococcus* endophthalmitis.

For testing antibiotic susceptibilities, proper guidelines do not exist for *Kocuria*. Hence, susceptibility breakpoints for *Staphylococci* are used instead. This can cause a misdiagnosis of either the sensitivity or the resistance pattern in these organisms. Savini et al. [1], in their report, discussed the implications of this misdiagnosis which can lead to recurrent infections and a morbid clinical outcome. It has also been shown that most pathogenic species are susceptible to broad-spectrum antibiotics. In the current reported series, similarly, six of eight cases of *Kocuria* endophthalmitis reported resolution of infection and inflammation at the last visit. Among the three that did not, two had recurrent retinal detachment and one had corneal decompensation which possibly added to the persistence of the inflammation. This relatively favorable outcome could be attributed to a favorable antibiotic susceptibility pattern as seen in Table 3.

The current study has a few inherent limitations. The effect of various confounding factors could not be independently assessed due to the retrospective nature of the study. The limited sample size did not allow us to assess any parameters that could have influenced the outcome. A proportion of cases of endophthalmitis in this series were post trauma. Trauma itself is a confounding factor for a final unfavorable visual outcome. Thus, it would be difficult to clearly delineate in the post-traumatic subset, whether the unfavorable visual outcome is due to trauma or due to the subsequent endophthalmitis. Though 75% cases in the current study had resolution of endophthalmitis at the last follow-up, visual improvement is limited due to anterior segment opacities. Further corneal intervention for those patients is pending as this manuscript is being written. Following corneal intervention, further visual improvement can be expected. In conclusion, *Kocuria* endophthalmitis is a relatively rare but emerging cause of endophthalmitis and is often misdiagnosed as *CoNS* endophthalmitis. The occurrence of *Kocuria* spp. in patients with endophthalmitis should not be ignored as contaminants. Though clinical presentation can often be variably late, timely and appropriate management with multiple interventions as and when required can result in an acceptable visual and anatomic outcome.
